# Two adult 46,XY sisters with female-like phenotype due to a novel homozygous *LHCGR* splice-site variant

**DOI:** 10.1210/jcemcr/luag155

**Published:** 2026-06-09

**Authors:** Marcela G Meirelles, Renata C Scalco, Juliana M Marques, José Lima Junior, Cristina F Bueno, Berenice B Mendonca

**Affiliations:** Unidade de Endocrinologia do Desenvolvimento, Laboratório de Hormônios e Genética Molecular LIM/42, Laboratório SELA, Hospital das Clínicas da Faculdade de Medicina da Universidade de São Paulo (HC-FMUSP), São Paulo 05403-000, Brazil; Unidade de Endocrinologia do Desenvolvimento, Laboratório de Hormônios e Genética Molecular LIM/42, Laboratório SELA, Hospital das Clínicas da Faculdade de Medicina da Universidade de São Paulo (HC-FMUSP), São Paulo 05403-000, Brazil; Unidade de Endocrinologia Genética (LIM/25), Disciplina de Endocrinologia, Departamento de Clínica Médica, Hospital das Clínicas da Faculdade de Medicina da Universidade de São Paulo (HC-FMUSP), São Paulo 01246-903, Brazil; Unidade de Endocrinologia do Desenvolvimento, Laboratório de Hormônios e Genética Molecular LIM/42, Laboratório SELA, Hospital das Clínicas da Faculdade de Medicina da Universidade de São Paulo (HC-FMUSP), São Paulo 05403-000, Brazil; Divisão de Endocrinologia, Departamento de Clínica Médica, Faculdade de Ciências Médicas, Irmandade da Santa Casa de Misericórdia de São Paulo, São Paulo 01221-020, Brazil; Divisão de Endocrinologia, Departamento de Clínica Médica, Faculdade de Ciências Médicas, Irmandade da Santa Casa de Misericórdia de São Paulo, São Paulo 01221-020, Brazil; Unidade de Endocrinologia do Desenvolvimento, Laboratório de Hormônios e Genética Molecular LIM/42, Laboratório SELA, Hospital das Clínicas da Faculdade de Medicina da Universidade de São Paulo (HC-FMUSP), São Paulo 05403-000, Brazil

**Keywords:** differences of sex development, luteinizing hormone receptor, leydig cell hypoplasia, splice-site mutation, 46,XY DSD

## Abstract

The luteinizing hormone/choriogonadotropin receptor (LHCGR) is essential for Leydig cell function, gonadal steroidogenesis, and male sexual differentiation. Loss-of-function variants in *LHCGR* are a rare cause of 46,XY differences of sex development (DSD). We report 2 46,XY adult sisters born to consanguineous parents, who presented with primary amenorrhea and absent secondary sexual characteristics. Both exhibited tall stature, female external genitalia, hypergonadotropic hypogonadism with very low testosterone levels. Imaging revealed bilateral cryptorchid testes and absence of Müllerian structures. Both sisters underwent bilateral gonadectomy, and histopathological findings provide strong morphological support for Leydig cell hypoplasia. Genetic testing identified a novel homozygous splice-site variant (c.384-2A>G) in *LHCGR*, predicted to cause aberrant splicing and a loss of receptor function. These findings expand the mutational spectrum of *LHCGR*-related DSD, a rare cause of 46,XY DSD.

## Introduction

The luteinizing hormone/choriogonadotropin receptor (LHCGR, MIM:152790) plays a key role in human reproductive physiology by mediating luteinizing hormone (LH) and human chorionic gonadotropin signaling [1-4]. Pathogenic variants in *LHCGR* cause partial or complete resistance to LH signaling, resulting in a broad spectrum of reproductive phenotypes depending on sex, zygosity, and residual receptor function [3, 5-7]. In 46,XY individuals, biallelic loss-of-function variants lead to Leydig cell hypoplasia, characterized by impaired testosterone production and disorders of sex development [1, 2, 7, 8].

Although several *LHCGR* variants have been described, the identification of novel mutations and their clinical and molecular characterization remain important for understanding the phenotypic spectrum of this condition. Here, we report 2 adult siblings with a female phenotype and 46,XY karyotype caused by a novel homozygous splice-site variant in *LHCGR*.

## Case presentation

Two Caucasian 46,XY sisters, aged 31 years (Case 1) and 27 years (Case 2), born to consanguineous parents and raised as females, presented with primary amenorrhea and absence of secondary sexual characteristics. The patients were recruited as part of an ongoing research project investigating disorders of sex development (DSD) at Hospital das Clínicas, Faculdade de Medicina da Universidade de São Paulo. Both sisters were assigned female sex at birth and consistently identified as women.

### Case 1

Physical examination revealed a height of 184 cm (target height, 173 cm), body weight of 118 kg, and a body mass index of 34.8 kg/m^2^. Breast development was Tanner stage I, pubic hair Tanner stage IV, and the external genitalia appeared typically female (Sinnecker score, 5). She also had class I obesity, type 2 diabetes mellitus, systemic arterial hypertension, and dyslipidemia.

### Case 2

The 27-year-old sister had a similar clinical presentation, with a height of 188 cm, Tanner stage I breast development, Tanner stage IV pubic hair, and typically female external genitalia (Sinnecker score, 5).

## Diagnostic assessment

### Case 1

Laboratory evaluation demonstrated hypergonadotropic hypogonadism, characterized by elevated gonadotropin levels, with predominance of LH levels, very low serum testosterone, and undetectable estradiol levels (Table 1). She also had altered metabolic parameters, including fasting glucose of 111 mg/dL (SI: 6.2 mmol/L) (reference range, 70-99 mg/dL [SI: 3.9-5.5 mmol/L]), glycated hemoglobin (HbA1c) of 6.0% (SI: 42 mmol/mol) (reference range, <5.7% [SI: <39 mmol/mol]), low-density lipoprotein cholesterol of 128 mg/dL (SI: 3.3 mmol/L) (recommended target, <100 mg/dL [SI: <2.6 mmol/L]), triglycerides of 185 mg/dL (SI: 2.1 mmol/L) (reference range, <150 mg/dL [SI: <1.7 mmol/L]), and high-density lipoprotein cholesterol of 33 mg/dL (SI: 0.9 mmol/L) (reference range, >40 mg/dL [SI: >1.0 mmol/L]), consistent with obesity-related metabolic dysfunction.

**Table 1:** Serum hormonal levels in the 2 46,XY sisters

Hormones	Case 1 (31 years)	Case 2 (27 years)	Reference range (adult male)
LH	49-60 mIU/mL (49-60 IU/L)	52-58 mIU/mL (52-58 IU/L)	0.6-12 mIU/mL (0.6-12 IU/L)
FSH	46-50.7 mIU/mL 46-50.7 IU/L	47-49 mIU/mL (47-49 IU/L)	0.9-12 mIU/mL 0.9-12 IU/L
Estradiol	<20 pg/mL (<73.4 pmol/L)	<20 pg/mL (<73.4 pmol/L)	≤44 pg/mL (≤162 pmol/L)
Total testosterone	<10 ng/dL (<0.35 nmol/L)	<10 ng/dL (<0.35 nmol/L)	254-803 ng/dL (8.8-27.8 nmol/L)
Progesterone	<0.5 ng/mL (<1.6 nmol/L)	<0.5 ng/mL (<1.6 nmol/L)	<0.5 ng/mL (<1.6 nmol/L)

Abnormal values are shown in bold font. Values in parentheses are Système International (SI) units.

Abbreviations: FSH, follicle-stimulating hormone; LH, luteinizing hormone.

Transabdominal pelvic ultrasound and pelvic magnetic resonance imaging (MRI) demonstrated absence of Müllerian structures. A left inguinal testis measuring 2.6 × 1.4 × 3.3 cm (volume 6.2 cm^3^) (Fig. 1A and 1B) and a right intra-abdominal testis measuring 3.2 × 2.5 × 1.7 cm (volume 7.0 cm^3^) (Fig. 1C) were identified. Both gonads exhibited regular morphology and homogeneous signal intensity. Cytogenetic analysis revealed a 46,XY karyotype.

**Figure 1:**
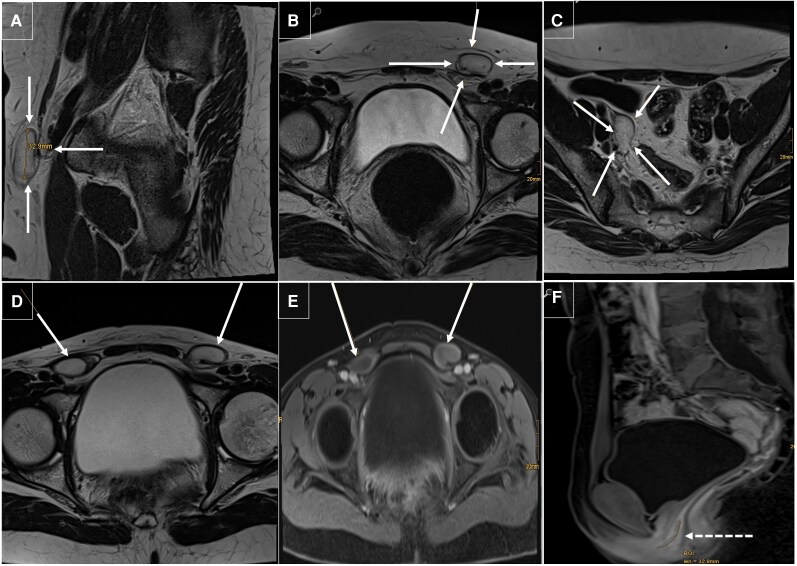
Pelvic magnetic resonance imaging (MRI) findings in 2 sisters with 46,XY disorder of sex development. (A–B) Case 1: Pelvic MRI demonstrates a left inguinal testis (solid white arrows) measuring 2.6 × 1.4 × 3.3 cm (volume 6.2 cm^3^), with regular morphology and homogeneous signal intensity. (C) Case 1: Right intra-abdominal testis (solid white arrows) measuring 3.2 × 2.5 × 1.7 cm (volume 7.0 cm^3^). (D–E) Case 2: Pelvic MRI shows bilateral inguinal testes (solid white arrows) without structural abnormalities; the right gonad measures 3.9 × 2.2 × 1.2 cm (volume 5.3 cm^3^) and the left gonad measures 3.9 × 2.6 × 1.2 cm (volume 6.3 cm^3^). (F) Case 2: Blind-ending vaginal pouch measuring 3.3 cm in length (dashed white arrow) with no visualization of Müllerian structures.

### Case 2

This sister also exhibited hypergonadotropic hypogonadism with markedly reduced testosterone levels (Table 1). Pelvic MRI demonstrated bilateral inguinal testes. The right gonad measured 3.9 × 2.2 × 1.2 cm (volume 5.3 cm^3^) and the left gonad measured 3.9 × 2.6 × 1.2 cm (volume 6.3 cm^3^), with no structural abnormalities identified (Fig. 1D and 1E). A blind-ending vaginal pouch measuring 3.3 cm in length was also observed (Fig. 1F), with the absence of Müllerian structures. Cytogenetic analysis also demonstrated a 46,XY karyotype.

Both patients underwent bilateral gonadectomy following diagnosis and psychological evaluation. On gross examination, the gonadal specimens demonstrated testes of reduced volume, with a smooth, glistening external surface, attached epididymis, and segments of the spermatic cord. On the cut section, the parenchyma appeared spongy and brownish. Microscopic examination of both testes revealed markedly atrophic testicular parenchyma, with numerous seminiferous tubules showing prominent peritubular hyalinization. Germ cells were present up to the stage of primary spermatocytes, without evidence of complete spermatogenesis. The interstitial compartment showed areas of fibrosis with extremely rare Leydig cells, consistent with severe Leydig cell hypoplasia (Fig. 2A and 2B). Immunohistochemical analysis further supported these findings, with inhibin and calretinin positivity detected only in rare, scattered interstitial foci corresponding to rudimentary Leydig cell structures, underscoring the marked impairment of Leydig cell differentiation.

**Figure 2:**
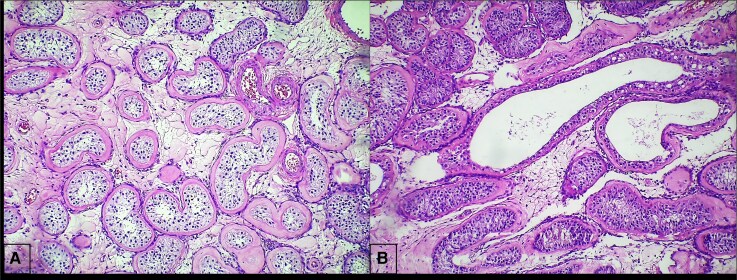
Representative hematoxylin and eosin–stained sections of gonadal tissue at 40× magnification. (A) Case 1: Right intra-abdominal testis showing markedly atrophic testicular parenchyma, with numerous seminiferous tubules exhibiting prominent peritubular hyalinization and reduced tubular lumina. Germ cells are present up to the stage of primary spermatocytes, without evidence of complete spermatogenesis. The interstitial compartment shows fibrotic areas with extremely rare Leydig cells, consistent with severe Leydig cell hypoplasia. (B) Case 1: Left inguinal testis demonstrate similar architectural alterations, with relatively better preservation of spermatogenic activity compared with the intra-abdominal gonad, although still incomplete. Scale bar = 50 μm.

Genetic and *in silico* analyses were performed to further characterize the patients' phenotype. For Case 1, next-generation sequencing performed on peripheral blood using a targeted 46,XY DSD gene panel identified the novel homozygous *LHCGR* splice-site variant (NM_000233.4:c.384-2A>G) and was classified as pathogenic according to American College of Medical Genetics and Genomics criteria (PM2, PVS1, PP4). In Case 2, the same variant was confirmed by Sanger sequencing (Fig. 3A). Unfortunately, the parents lived far from São Paulo and could not be reached for segregation analysis.

**Figure 3:**
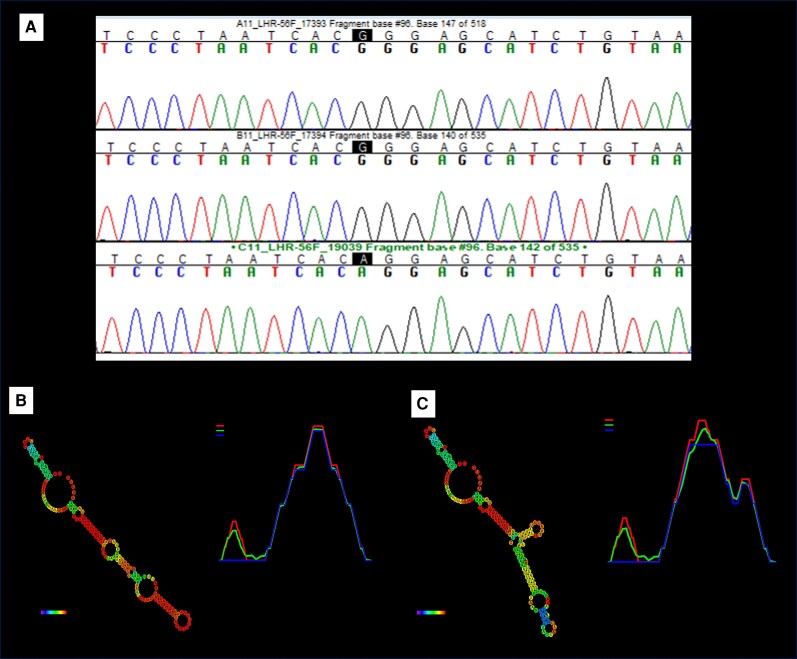
Molecular and *in silico* characterization of the *LHCGR* c.384-2A>G splice-site variant. (A) Representative Sanger sequencing electropherograms demonstrating the homozygous adenine-to-guanine substitution at the −2 position of intron 4 (c.384-2A>G; NM_000233.4), affecting the canonical splice acceptor site. The variant is identified by a single, unambiguous nucleotide peak. (B) Predicted RNA secondary structure of the wild type *LHCGR* sequence generated using ViennaRNA Fold. (C) Predicted RNA secondary structure of the mutant *LHCGR* sequence harboring the c.384-2A>G variant. Compared with the wild-type sequence, the mutant RNA structure exhibits reduced thermodynamic stability, a lower frequency of the minimum free energy conformation, and increased structural diversity, reflecting greater conformational heterogeneity. These alterations support impaired splicing efficiency and reduced transcript stability, consistent with aberrant *LHCGR* splicing and loss of receptor function.

*In silico* splice-site prediction tools indicated complete loss of the canonical AG acceptor site at intron 4. SpliceAI demonstrated a high probability of splice-site loss (Δ score 0.99), and MaxEntScan showed a marked collapse in splice-site strength, together suggesting aberrant splicing through exon skipping or intron retention. Ribonucleic acid (RNA) secondary structure modeling using ViennaRNA Fold further demonstrated reduced thermodynamic stability of the mutant transcript, as reflected by a less negative free energy (ΔG), a markedly lower frequency of the minimum free energy structure, and increased structural diversity compared with the wild-type sequence (Table 2, Fig. 3B and 3C).

**Table 2:** RNA secondary structure metrics comparing wild-type and mutant (c.384-2A>G) sequences predicted using ViennaRNA fold

Parameter	Wild type	Mutant (c.384-2A>G)	Data interpretation
Free Energy (ΔG)	−32.6 kcal/mol	−30.05 kcal/mol	The mutant shows reduced stability (less negative ΔG), suggesting structural destabilization.
MFE frequency	1.96%	0.60%	The lowest-energy structure is less prevalent in the mutant.
Diversity	22.99	35.29	The mutant exhibits a greater number of alternative conformations, leading to increased structural heterogeneity.

Abbreviations: ΔG, Gibbs free energy; MFE, minimum free energy.

The diagnosis was challenging due to the discordance between the female phenotype and the 46,XY karyotype, requiring integration of clinical, hormonal, imaging, and genetic findings.

## Treatment

Both patients were diagnosed with 46,XY DSD due to a homozygous pathogenic *LHCGR* variant and underwent prophylactic bilateral gonadectomy. Hormone replacement therapy was initiated with oral estradiol valerate 2 mg daily. Given the presence of reduced bone mineral density, management prioritized analyzing the effect of estrogen and vitamin D and calcium replacement therapy before consideration of antiresorptive treatment. Case 1 also required management of metabolic comorbidities and was treated with metformin 2 g daily, atorvastatin 40 mg daily, ezetimibe 10 mg daily, losartan 50 mg daily, and fluoxetine 40 mg daily. She also received calcium carbonate 500 mg daily and cholecalciferol 14 000 IU weekly to support bone health. Case 2 also received adjunctive vaginal estriol. Psychological support was provided.

## Outcome and follow-up

At the last follow-up in October 2025, both patients remained clinically stable and under regular endocrinology care. Bone evaluation demonstrated an improvement of bone mass in both cases, without a history of fragility fractures. Management focused on adequate estrogen replacement with annual reassessment of bone mineral density to guide future consideration of antiresorptive therapy. At follow-up, both sisters showed progressive breast development and a more typically female fat distribution after initiation of hormone therapy and reported satisfaction with body changes and improved psychosocial well-being. No adverse or unanticipated events were observed during follow-up.

## Discussion

We describe 2 adult 46,XY sisters with a female-like phenotype caused by a novel homozygous *LHCGR* splice-site variant leading to complete loss of LH receptor function. The clinical and biochemical profile was characterized by hypergonadotropic hypogonadism with markedly reduced testosterone levels, a hallmark of the LH signaling defects [2-4, 6-8]. In *LHCGR*-related disorders, LH resistance represents the primary endocrine abnormality, reflecting the inability of Leydig cells to respond to LH stimulation despite markedly elevated circulating LH levels [1-4, 8-14].

Although LH elevation is the defining biochemical feature of LHCGR dysfunction, both individuals also exhibited disproportionately elevated follicle-stimulating hormone concentrations. This finding likely reflects longstanding Sertoli cell damage secondary to chronic cryptorchidism, as supported by histological evidence of incomplete spermatogenesis and seminiferous tubule atrophy [1-3, 7, 8].

*In silico* analyses predicted disruption of the canonical splice acceptor site, leading to aberrant splicing and reduced RNA stability, findings consistent with complete loss of receptor function. Variants affecting this same acceptor region (c.384-2) have previously been reported in individuals with 46,XY DSD, highlighting the functional relevance of this splicing site for normal *LHCGR* expression and activity [12]. In addition, Zhang et al demonstrated that splice-site mutations at c.384-2 generate multiple abnormal transcripts and markedly impair receptor expression and downstream signaling in women with empty follicle syndrome [12]. Because the variant identified in our patients (c.384-2A>G) involves the same splice acceptor site, it is highly likely to act through a similar mechanism of splicing defect and receptor inactivation. Collectively, these observations reinforce that complete *LHCGR* loss of function produces a sex-dependent phenotypic spectrum, ranging from severe ovulatory dysfunction in 46,XX individuals [12] to Leydig cell hypoplasia with absent virilization in 46,XY patients. A recent systematic review on Leydig cell hypoplasia highlighted the low risk of germ cell malignancy in LHCGR receptor defects, supporting individualized decision-making regarding gonadal management [16].

Histological analysis of the testes showed seminiferous tubules with a delicate basal membrane and spermatogenesis up to the spermatocyte stage, which is a frequent finding in other cases of the complete form of LHCGR deficiency [14, 15]. The presence of epididymides is common in 46,XY females due to pathogenic variants in *LHCGR* [14], suggesting that some amount of testosterone is produced even in the complete form of the disorder and that the Wolffian ducts are more sensitive to androgen action than the external genitalia. We had the opportunity to measure testosterone levels in the testicular vein in one of the first cases we treated and obtained a value of 127 ng/dL (4.4 nmol/L), compared with 20 ng/dL (0.7 nmol/L) in peripheral blood [16]. Normal basal progesterone levels observed in our patients are also relevant in the differential diagnosis, as they exclude 17α-hydroxylase deficiency, a relatively frequent form of congenital adrenal hyperplasia in Brazil, typically associated with elevated progesterone levels and low androgen production [17].

Adults with hypogonadism due to complete loss of gonadal steroidogenesis require lifelong sex hormone replacement therapy to support somatic and psychological wellbeing, optimize bone mineral density, and maintain cardiovascular and metabolic health. Hormone replacement regimens should aim to approximate physiological patterns across the lifespan. In 46,XY individuals with intra-abdominal testes, careful consideration of gonadal management is warranted given the increased risk of gonadal malignancy associated with Y chromosome material, with decisions individualized according to phenotype, age, risk profile, and patient preferences within a multidisciplinary care framework [6, 9, 13, 14].

This report has some limitations. Functional studies to confirm the effect of the identified variant were not performed, and parental segregation analysis was not available. In addition, RNA studies were not conducted to directly confirm the predicted splicing defect.

In conclusion, the concordant clinical, hormonal, histopathological, and *in silico* findings establish a clear genotype–phenotype correlation and provide a mechanistic explanation for Leydig cell hypoplasia and severe testosterone deficiency in our patients. This report expands the mutational spectrum of *LHCGR*-related 46,XY DSD and highlights the value of integrating molecular and tissue-level analyses in the evaluation of these rare LH signaling defects.

## Learning points

- LHCGR loss-of-function variants are a rare cause of 46,XY DSD.
- Homozygous splice-site variants may lead to complete Leydig cell hypoplasia.
- Genetic testing is essential in 46,XY individuals with female phenotype and hypergonadotropic hypogonadism.

## Data Availability

Original data generated and analyzed for this case report are included in this published article.

## Abbreviations

DSD: differences of sex development
FSH, FSH: follicle-stimulating hormone
LH: luteinizing hormone
LHCGR: luteinizing hormone/choriogonadotropin receptor
ΔG: Gibbs free energy
MFE: minimum free energy
MRI: magnetic resonance imaging
